# Modulated Mechanical Properties of Epoxy-Based Hybrid Composites via Layer-by-Layer Assembly: An Experimental and Numerical Study

**DOI:** 10.3390/polym16243559

**Published:** 2024-12-20

**Authors:** Hee-Chang Jeon, Young-Seong Kim

**Affiliations:** 1 Quantum Functional Semiconductor Research Center, Dongguk University, Jung-gu, Seoul 04620, Republic of Korea; hcjeon@dongguk.edu; 2 Department of Mechanical, Robotics and Energy Engineering, Dongguk University, Jung-gu, Seoul 04620, Republic of Korea

**Keywords:** layer-by-layer casting, epoxy-based composite, hydroxyapatite, cellulose nanocrystal, finite element method

## Abstract

In this study, epoxy-based composites were fabricated using a layer-by-layer assembly technique, and their mechanical properties were systematically evaluated. The inclusion of cellulose nanocrystals led to variations in the mechanical properties of the composites. These modified properties were assessed through tensile and flexural tests, with each layer cast to enhance strength. Due to the inherent characteristics of epoxy, a single specimen was fabricated through chemical bonding, even post-curing. This approach demonstrated that a three-layer structure, developed using the layer-by-layer method, exhibited improved elastic and flexural moduli compared to a single-layer composite. This improvement aligns with theoretical predictions, which suggest that stiffness increases when stiffer materials are positioned farther from the neutral axis in a layered structure. Furthermore, numerical analysis validated changes in stress distribution across each layer. Consequently, this method enables the production of composites with superior mechanical properties while minimizing the quantity of cellulose nanocrystals required.

## 1. Introduction

Polymer-based hybrid and composite materials have been extensively studied for their potential to enhance mechanical properties and biomedical characteristics, particularly those composites that incorporate hydroxyapatite (HAP) [1,2]. HAP is known for its superior mechanical properties, including compressive strength, flexural strength, and elastic modulus [3,4,5], often surpassing those of cortical bones [5,6,7]. The hierarchical structure of bone imparts unique mechanical properties that combine strength and toughness [8]. Replicating the strength and characteristics of bone in biomimetic structures, particularly those with complex shapes and varying mechanical properties, presents significant challenges. Consequently, new approaches are being explored to fine-tune mechanical properties through compositional changes in additives, stimulating further research on layer-by-layer (LbL) assembly. This technique has been employed to develop HAP-based materials for pediatric traumatology and orthopedics [9,10].

Recently, interest in multi-layer structured composites has increased to achieve desired mechanical strengths. For example, cellulose nanofibers (CNFs) have demonstrated improved strength in polysaccharide films produced using both blending and LbL methods, depending on the matrix [11]. In polymer nanocomposite fabrication, LbL casting has proven that while the matrix remains constant, the strategic addition of various fillers enhances the energy density of multi-layer structures compared to single-layer composites [12]. Multi-layer composites created through LbL assembly facilitate the engineering of material functionality, enabling their application across a broad spectrum of applications.

The LbL assembly technique involves alternating immersion in different composite solutions to systematically modify properties, construct layers, and enable functional manufacturing. This process typically involves hydrogen bonding, covalent interactions, and hydrophobic interactions [13].

Recent research has extensively explored enhancing the mechanical strength of hybrid epoxy-based composites via tensile, flexural, and impact testing. Notably, considerable focus has been placed on the influence of stacking sequences on the strength and variability in epoxy composites [14,15]. Such hybridization utilizes the benefits of constituent materials while addressing their intrinsic weaknesses, resulting in the creation of superior materials [16]. However, the majority of these studies have focused on hybrid structures in laminated forms, predominantly using the hand lay-up technique, with limited investigation of the changes in mechanical properties in cast forms.

This study aims to enable the facile formation of large-area artificial bone with adjustable mechanical properties using the LbL assembly technique. This approach is expected to have broad applications in the uniform synthesis or mixing of functional substances, natural materials, and additives, particularly at high concentrations. Contrary to single-layer composites, this study involves the fabrication of laminated, particulate-reinforced materials using the LbL casting approach to produce hybrid multi-layer composites. The differences in the mechanical properties of these composites are analyzed through both experimental testing and simulation, confirming the enhanced characteristics of the hybrid composite structure. Notably, while the usage of nanocellulose is reduced compared to traditional methods, the mechanical properties are improved through the LbL assembly technique, surpassing the mere stiffness enhancements typically achieved by direct nanocellulose addition. Furthermore, the decreased incorporation of nanocellulose crystals leads to lower production costs. Despite the reduced content of additives, the three-layer structure shows an increase in both elastic and flexural moduli via this integrated approach. Although the tensile test indicates an improvement in the modulus, the typical brittleness induced by cellulose addition is effectively reduced. Variations in the properties of the epoxy-based composites owing to the LbL assembly are analyzed by comparing the experimental findings with simulations using the finite element method (FEM).

## 2. Materials and Methods

### 2.1. Materials

The epoxy and hardener used in this study were 105 epoxy resin and 207 hardener, respectively, from West System Co. (Bay City, MI, USA). HAP, with a purity of 90% or greater (as Ca_3_(PO_4_)_2_, KT) was purchased from Sigma–Aldrich (St. Louis, MO, USA). The nanocellulose used was cellulose nanocrystals (CNCs) obtained from the Process Development Center at the University of Maine. For specimen preparation, silicone molds were fabricated using Mold Max 30 from Smooth-On, Inc. (Macungie, PA, USA).

### 2.2. Preparation of Nanocellulose

CNCs were initially in a 10.4 wt% slurry and were converted into powder by freeze-drying. This process was performed using a freeze dryer (FD8508 model, ilShinBioBase Co., Ltd. (Dongducheon-si, Republic of Korea)) over 3 d.

### 2.3. Preparation of Composite Materials

First, HAP was vacuum-dried at 100 °C for 30 min and weighed according to the required wt% for drying. Subsequently, the hardener was added, and the mixture of hardener and HAP powder was ultrasonicated for 30 min to ensure thorough mixing. After the ultrasonic treatment, the resin was added and stirred using a wooden stick. To remove air bubbles, the mixture underwent vacuum treatment at room temperature before being poured into prepared silicone molds shaped for the specimens and left to cure. High-temperature curing was performed at 80 °C for 3 h, followed by mechanical property testing. The hardener and resin were mixed at a weight ratio of 1:3. CNCs were added to the hardener and HAP mixture, followed by an additional 30 min of ultrasonication.

**Figure 1:**
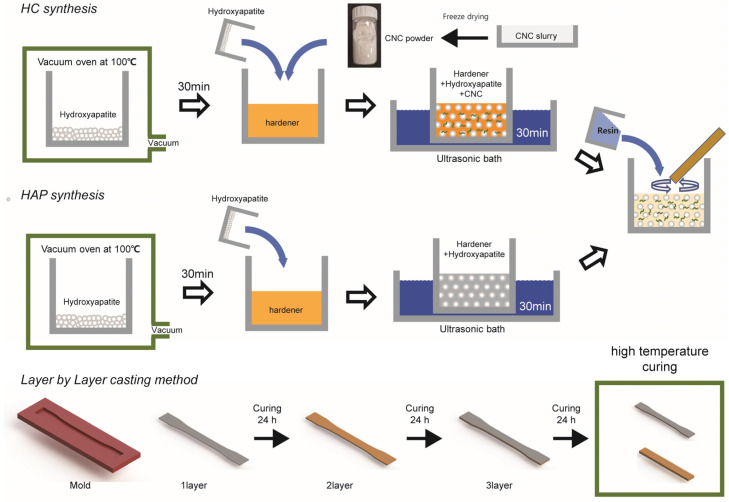
Schematic diagram illustrating the preparation of composite materials.

For single-layer fabrication, specimens comprising 5 wt% HAP and 5 wt% HAP with 1 wt% CNC were cured, demolded after one day, and subjected to high-temperature curing at 80 °C. For LbL assembly, each layer was cured at room temperature for one day before the subsequent layer was applied, with final high-temperature curing at 80 °C after all layers were completed. Each of the three layers was prepared to be one-third of the ASTM standard [17,18] thickness for tensile and flexural test specimens. Five specimens of each type were fabricated and analyzed (Figure 1).

### 2.4. Materials Characterization

Tensile tests were conducted according to ASTM D638 standards [17] using Type 1 specimen shapes, with measurements recorded at a feed rate of 5 mm/min. For the flexural tests, ASTM D790 standards [18] were followed, with specimens prepared to a length of 12 mm and a specified width and thickness. The three-point bending test had a span of 72 mm, and measurements were recorded at a feed rate of 1.96 mm/min, as specified by the standard. Tests were conducted using a universal testing machine (Oriental TM Co., Ltd., (Siheung-Si, Republic of Korea) OTU-2).

For the FEM analysis, modeling was performed using Abaqus/CAE 2020 (Dassault systems, (Vélizy-Villacoublay, France)). The material properties for the simulations were based on experimental data from single-layer specimens. An explicit dynamic analysis method was employed.

## 3. Result and Discussion

**Figure 2:**
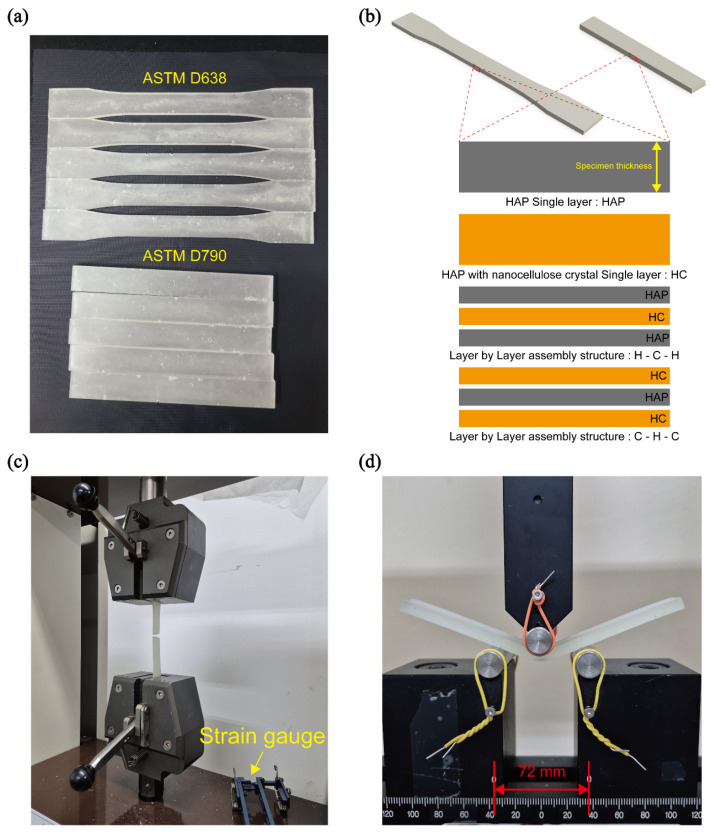
(**a**) Tensile and flexural specimens prepared in accordance with ASTM standards. (**b**) Schematic representation of both single-layer and LbL structures. (**c**) Tensile test. (**d**) Flexural test.

To analyze changes in the mechanical properties due to the addition of HAP and CNC to epoxy, comparative analyses were conducted between single-layer structures [19] and LbL assemblies. Tensile and flexural specimens were prepared using silicone molds fabricated according to ASTM standards [17,18] (Figure 2a). Single-layer specimens containing 5 wt% HAP were designated as HAP, while specimens containing 5 wt% HAP with 1 wt% CNC were designated as HC. The specimens fabricated as three-layer composites in the HAP/HC/HAP and HC/HAP/HC configurations were denoted as HCH and CHC, respectively. These layers were constructed according to the specified specimen thickness, with each layer cured individually for the three-layer composites (Figure 2b).

Tensile tests were conducted in accordance with ASTM standards [17], employing a strain gauge with a gauge length of 50 mm (Figure 2c). Flexural tests were also performed following ASTM standards [18] (Figure 2d).

**Figure 3:**
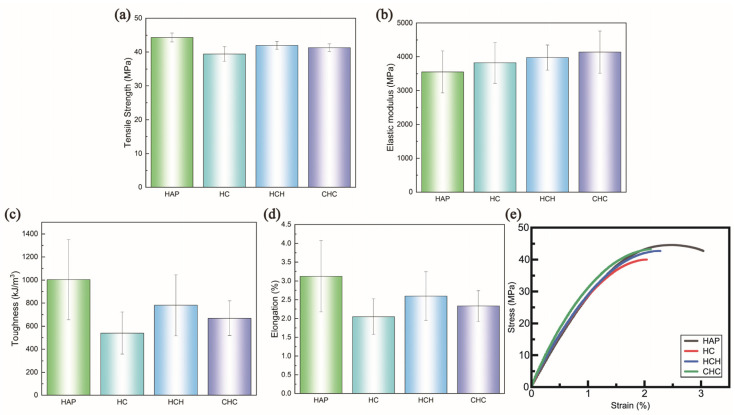
Experimental data from tensile tests showing (**a**) tensile strength, (**b**) elastic modulus, (**c**) toughness, (**d**) elongation, and (**e**) stress–strain curve.

In the tensile tests, the strength values across the specimens were broadly similar, with the single-layer HAP having the highest strength, although the differences were not statistically significant (Figure 3a). However, variations in the elastic modulus were more pronounced. Consistent with previous studies [19], the addition of CNC was found to increase the elastic modulus, a trend that was also observed in this study. Notably, specimens constructed in a three-layer configuration showed a more substantial increase in the modulus, particularly in the CHC configuration.

Despite the lower CNC weight ratio compared to the single-layer specimens, the CHC structure demonstrated enhanced stiffness, with an increase of 8.34% [19]. This increase was attributed to the formation of transcrystals in the CNC-reinforced brittle layer, which proved to be stiffer and more brittle than conventional crystals [20]. This effect was further validated by scanning electron microscopy (SEM) images, which revealed distinct crystal formations emerging from the curing process when CNCs were incorporated [21]. The positioning of the CNC-reinforced layer, either in the center or on the outer layers, facilitated the tuning of mechanical properties, confirming that simple adjustments in structural design could optimize mechanical characteristics. While not as significant as in the CHC structure, the HCH structure showed an increase in stiffness over the HC configuration by 4.09% (Figure 3b) [14].

Furthermore, previous studies have shown that using only the CNC structure significantly reduces toughness and elongation. However, this limitation was reduced in the LbL assembly, as evidenced by the enhanced toughness and elongation observed. In the HCH structure, elongation improved by 26.6% compared to the HC structure, while the CHC configuration exhibited a 13.7% increase. Toughness was also notably enhanced, improving by 44.7% in the HCH structure and by 23.9% in the CHC structure (Figure 3c,d). These improvements are further highlighted in the measured stress–strain curve data (Figure 3e).

**Figure 4:**
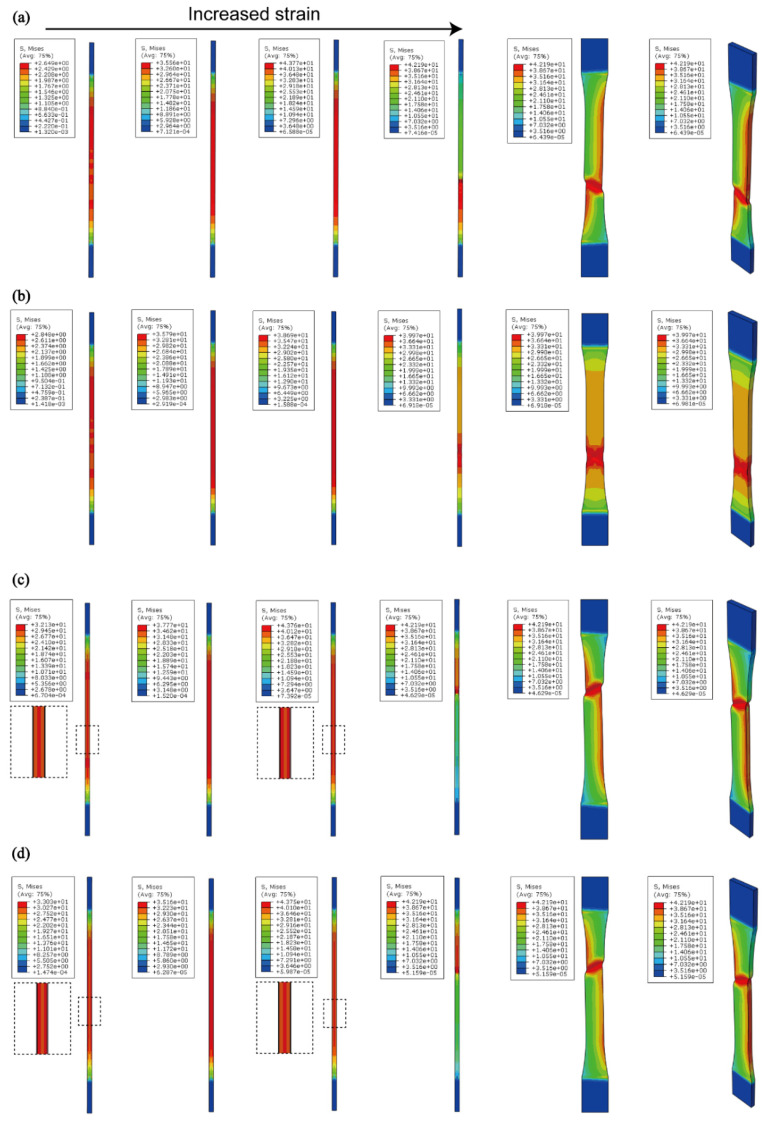
Numerical simulation results of the tensile test for (**a**) HAP, (**b**) HC, (**c**) HCH, and (**d**) CHC structures.

When the LbL assembly was designed with a brittle/ductile/brittle configuration, the elastic modulus increased to approximately 4 GPa. The HCH structure exhibited a higher elastic modulus than the single-layer configuration while demonstrating superior toughness compared to CHC (Figure 3b,d). This distinction in properties was further revealed through the stress distribution and propagation patterns observed in the FEM tensile test results. These results revealed significant differences in stress distribution between the multi-layer and single-layer structures (Figure 4). Notably, a comparison of stress concentrations during the initial loading phase and just prior to fracture highlighted distinct trends in the three-layer specimens. The HCH and CHC specimens exhibited opposite stress distribution trends.

In the early stages of strain, stress was concentrated in the CNC layer; however, in the later stages, it propagated to the HAP layers, with a tendency toward localization rather than widespread distribution. This behavior explained why fracture in the single-layer specimens occurred in the lower region, whereas in the three-layer structures, it shifted to the upper region.

In the three-layer structure, the CNC layer exhibited stress concentration under initial loading, whereas the HAP layer demonstrated stress concentration under higher loads (Figure 4c,d). This observation was consistent with previous findings for multi-layer systems composed of ductile and brittle materials, where stress gradients shifted to the brittle layer, surpassing the fracture limit more effectively than in single-layer configurations. The interactions between layers were modified by stress-inhibiting forces that influenced the necking direction, altering the stress components of the two layers and, ultimately, the fracture strain [22]. Consequently, the CNC-containing layer, characterized by lower ductility and elongation, experienced an initial stress concentration and transferred the load to the non-CNC layers [23].

Flexural stress and the flexural modulus were calculated as [24]

$$\sigma_{f}=\frac{3PL}{2bd^{2}},$$

$$E_{f}=\frac{L^{3}m}{4bd^{3}},$$

where *P* denotes the force applied during the flexural test, *L* is the span, *b* is the specimen width, and *d* is the specimen depth. In the calculation of the flexural modulus, *m* represents the slope of the initial load–displacement curve.

**Figure 5:**
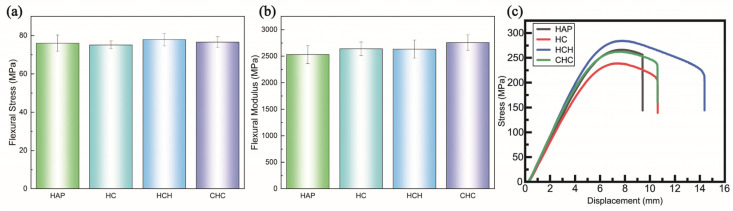
Experimental data from flexural tests detailing (**a**) flexural stress, (**b**) flexural modulus, and (**c**) stress–displacement curve.

In the flexural tests, although differences in flexural strength were minimal, the CHC three-layer structure demonstrated the highest flexural modulus, marking a 4.46% increase compared to the HC single layer. This result confirmed that the hard–soft–hard LbL assembly achieved the highest flexural modulus (Figure 5). Achieving effective bonding or adhesion strength in such soft–hard multi-material structures remains a considerable challenge, primarily due to the lack of inherent chemical affinity [25,26]. Previous studies have shown that in polymer-based soft–hard multi-layer structures, the flexural modulus often decreases compared to that in single-layer configurations [27]. However, when a multi-layer structure is crafted using the same epoxy matrix as CNFs to induce mechanical property variations, chemical bonding between the layers occurs. This interlayer chemical bonding significantly enhances the mechanical properties, particularly the flexural modulus, of the multi-layer configurations [11]. The flexural modulus is particularly sensitive to interfacial debonding, which can significantly affect its value [28].

In this study, no debonding was observed in the epoxy matrix, which facilitated the absorption of surface layer stress and resisted deformation in the middle layer through the LbL assembly. This configuration led to an increase in the flexural modulus despite the lower CNC content. The improvement was attributed to an increase in the inelastic component, driven by the enhanced ductility of the material [29]. The higher flexural modulus exhibited by the CHC composite in the LbL assembly was consistent with theoretical findings, which indicate that stiffness increases as layers with higher elastic moduli are positioned farther from the neutral axis, where stress is zero [30]. In the HCH structure of the LbL assembly, the CNC-containing layers, with their higher elastic modulus, were located near the neutral axis, the region of zero stress within the cross-section. Although these layers experienced relatively lower stress at the center of the beam, their high elastic modulus significantly improved resistance to both compression and tension in the bonded interlayers. As ductility increased, the inelastic component contributed to a greater total energy absorption.

This hybrid effect, arising from differences in deformation, influenced the interlayer stress distribution. The findings were consistent with those from FEM simulations. Furthermore, previous studies have demonstrated that the modulus of laminated hybrid composites can exceed that of single-material structures when layers with relatively lower stiffness are placed on the exterior and those with higher stiffness are positioned in the middle [29]. These results highlight the unique structural benefits of LbL assembly and demonstrate the potential for engineering composites with tunable mechanical properties that outperform traditional single-layer configurations.

**Figure 6:**
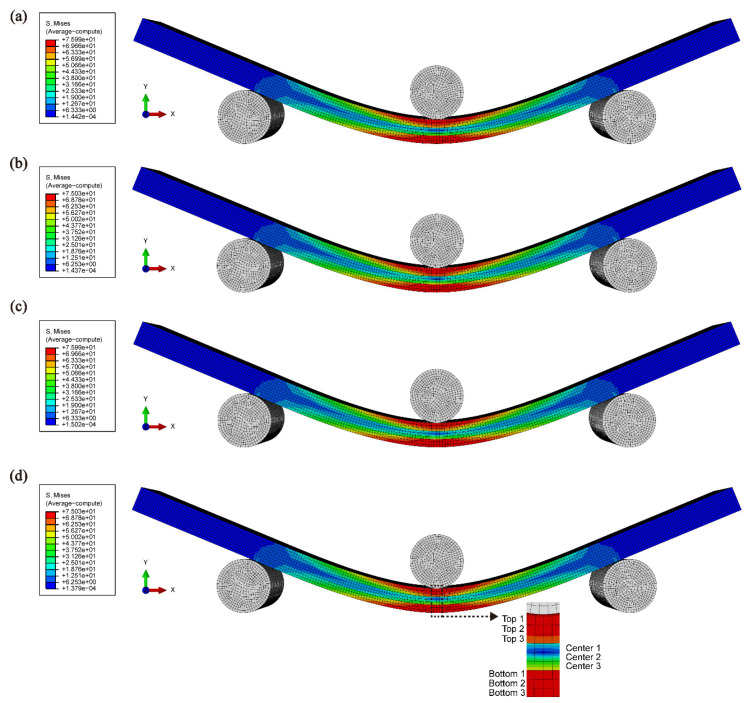
Numerical simulation of (**a**) HAP, (**b**) HC, (**c**) HCH, and (**d**) CHC at 10 mm displacement.

The FEM results revealed distinct stress distribution patterns across different samples. In the HAP sample, the middle layer exhibited a relatively low stress distribution (Figure 6a). In contrast, the HC sample demonstrated that the inclusion of a stiffer material with increased stiffness led to a relatively high stress concentration in the middle layer (Figure 6b). The HCH structure displayed behavior similar to that of the HAP sample in the force-receiving regions; however, high stress was concentrated in the upper regions of the CNC-containing layers, indicating reduced stress transfer to the middle layer (Figure 6c). Conversely, in the CHC structure, high stress was observed in the CNC-containing layers, with effective stress transfer extending to the middle layer, resulting in enhanced mechanical properties (Figure 6d).

These observations were numerically analyzed through simulations to evaluate the behavior of each element and layer, confirming significant differences in the behavior of single-layer and three-layer structures.

**Figure 7:**
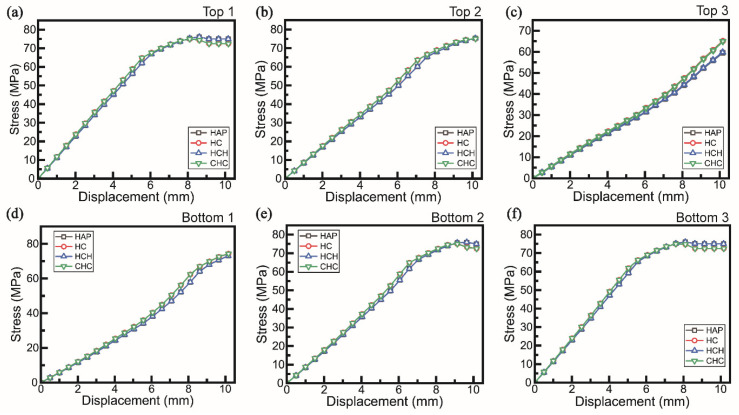
Numerical simulation graph of flexural test in (**a**–**c**) top layer and (**d**–**f**) bottom layer.

The LbL assembly was divided into top, center, and bottom layers, with elements analyzed at integrated points designated as Top 1, 2, and 3; Center 1, 2, and 3; and Bottom 1, 2, and 3, as shown in Figure 7, to evaluate stress–displacement behavior. Analysis of the top layer revealed elevated stress responses when the layer contained cellulose, with increased values observed for the LbL assembly (Figure 7a–c). The bottom layer exhibited similar stress variations in the presence of cellulose (Figure 7d–f).

In the bottom layer, specifically at Bottom 2 and 3, the CHC sample displayed a higher modulus at the initial slope compared to the HC sample, indicating changes in the modulus as tensile loads were applied to the bottom layer (Figure 7e,f). This difference highlighted how the incorporation of cellulose into the LbL assembly influenced the mechanical response and enhanced the modulus under tensile stress in specific layers.

In the center layer, a comparison of stress variations between the single-layer and LbL assembly revealed distinct trends depending on the composite structure of the LbL assembly (Figure 7a–c). This observation was particularly evident in the experimentally measured differences in the flexural modulus. In the HCH sample, the Center 1 element, which was connected to the top layer, exhibited the highest slope and stress, albeit marginally (Figure 7a). In Center 2, the stress response displayed contrasting characteristics compared to the single-material properties of the center layer. For the CHC sample, despite the absence of CNC in the center layer, its behavior closely resembled that of the HC sample due to multi-layer interactions. Conversely, the HCH sample, which included CNC in the center layer, exhibited behavior most similar to that of the HAP sample (Figure 7b).

At Center 3, the HCH sample exhibited the highest strength and slope up to a displacement of 4 mm. Beyond this point, the HC sample demonstrated higher values. At approximately 7 mm displacement, the stress and slope of the HCH and CHC samples intersected, after which the CHC sample exhibited greater stress and slope (Figure 7c). These results underscore the complex mechanical behavior of LbL-assembled composites, where interactions between layers significantly influence stress distribution and the modulus throughout the structure.

**Figure 8:**
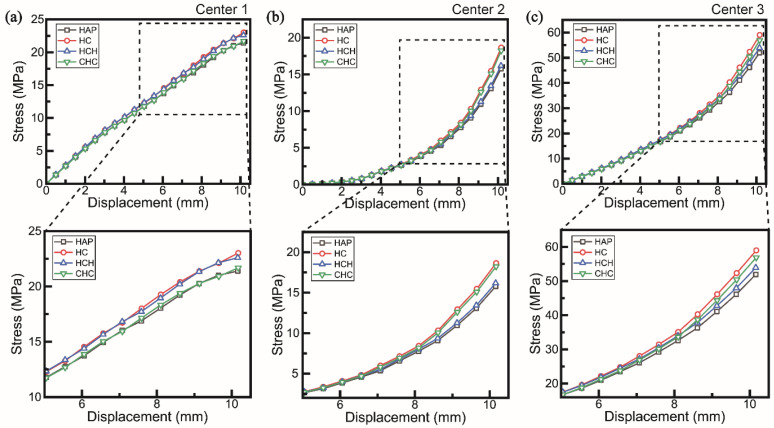
Numerical simulation graph of flexural test in (**a**–**c**) center layer.

The findings indicate that in LbL assemblies, samples such as CHC, where high-elastic-modulus layers are positioned externally, experience significant tensile and compressive stresses during flexural deformation. This resistance to deformation enhances the flexural modulus. The farther the elastic modulus layers are from the neutral axis, the greater the compressive and tensile stresses. The middle layer absorbs deformation and redirects the maximum stress to the outer high-elasticity layers, thereby increasing the overall flexural modulus. In samples such as HCH, the high elastic modulus of the middle layer indicates a tendency to absorb more stress, potentially leading to stress concentration within the middle layer. Additionally, interfacial stresses near the contact surfaces can localize stress concentration, with the high-modulus layer absorbing more load than the low-modulus layer, thereby influencing stress distribution (Figure 8) [30].

The simulation results demonstrate that composite materials containing only HAP assist in dispersing load through the outer layers due to their ductility [29]. Conversely, layers with high-modulus CNC positioned centrally minimize deformation and enhance resistance to compression and tension with increasing displacement, thereby increasing the overall flexural modulus of the beam.

**Figure 9:**
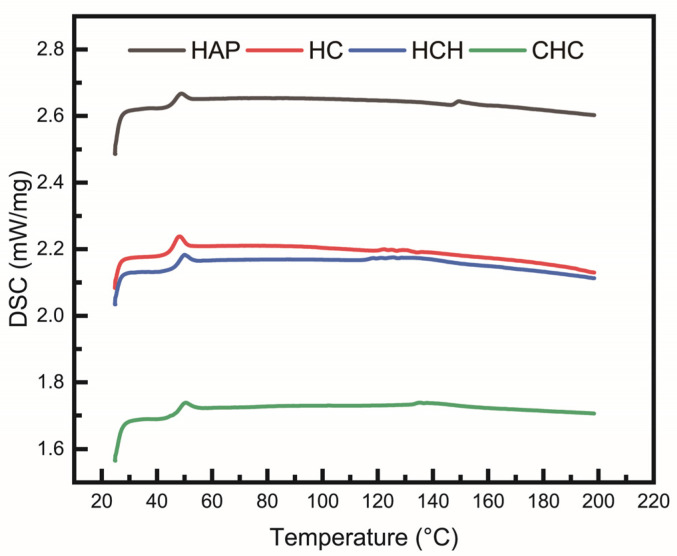
DSC curves of the epoxy composites.

From the differential scanning calorimetry (DSC) curve, changes in the glass transition temperature (Tg) can be observed. Specifically, a shift toward a higher Tg is evident in the three-layer structure compared to the single-layer structure. This increase is attributed to the enhanced physical cross-linking interactions between the layers in the three-layer structure, which restricts the movement of epoxy molecular chains, resulting in improved thermal stability (Figure 9) [31].

In conclusion, HAP-based composites with multi-layer structures absorb force over a smaller area, whereas HC-based composites distribute force over a wider area. This study successfully demonstrates the engineering of tensile and flexural modulus characteristics through multi-layer formation in epoxy-based composites. Compared to single-layer structures, LbL assembly incorporating CNC achieves enhanced mechanical properties while utilizing only one-third to two-thirds of the weight percent, highlighting its potential for creating advanced composites with improved performance.

The innovative LbL assembly offers significant advantages, including the enhancement of mechanical properties in brittle/ductile multi-layered composites. Stiffness and strength are optimized in a three-layer structure when the core thickness is less than half of the overall specimen thickness [30,32,33]. The superior mechanical properties of these multi-layered composites are primarily determined by the interfacial interactions between layers [34]. In brittle/tough systems, the tough layer effectively inhibits crack propagation from the brittle layer, resulting in a pronounced synergistic effect. Similarly, in PC/SAN multi-layer polymer composites, the brittle layer initially forms cracks at a specific deformation threshold, which induces shear band formation in the tough layer and mitigates tip stress at the cracks [35,36].

Moreover, the incorporation of nanocomposites into multi-layer structures enhances the elastic modulus due to transcrystal formation within the embedded layers, a phenomenon confirmed in multi-layered systems compared to single layers [37]. The formation of transcrystalline domains has been observed when CNCs are incorporated into polymers [38]. Previous studies have demonstrated that the mechanical properties of neat epoxy can be improved using HAP and CNCs, with CNCs specifically increasing stiffness, albeit at the expense of increased brittleness [19].

This study demonstrates that multi-layer composites produced through LbL assembly enable a reduction in filler weight percentage while achieving controlled enhancements in tensile modulus, toughness, and flexural modulus. Both experimental and simulation results confirm that these findings align with theoretical expectations, demonstrating that a range of mechanical properties can be tuned using the strategic design of multi-layer structures.

## 4. Conclusions

This study successfully developed a hybrid LbL assembly by combining HAP and epoxy-based composites, with and without CNC incorporation. The resulting structures demonstrated excellent mechanical properties, even at low CNC volume fractions. The results revealed that multi-layer structures, utilizing the mechanical property variations induced by CNC, could be efficiently fabricated into hybrid forms using the layer-by-layer casting method. This approach enabled precise control over tensile modulus, toughness, and flexural modulus within the LbL assembly. For the tensile modulus, interactions among the multi-layers induced changes in stress concentration by altering necking direction under strain. These effects were further influenced by material-specific stress variation characteristics associated with ductility. Analysis of the flexural modulus that the CHC configuration achieved the highest values, with numerical analysis confirming that stress distribution and deformation resistance were influenced by the elastic moduli of individual materials and their distances from the neutral axis. These findings validated the potential of LbL assembly to enhance both tensile and flexural mechanical properties. This study highlights the versatility of the LbL assembly method in tuning mechanical properties for a wide range of applications, such as large-area artificial bone synthesis. This approach is especially beneficial when the uniform mixing of additives is challenging or when high concentrations and consistent synthesis are difficult to achieve.

## Data Availability

The data presented in this study are available in the insert article.
